# Role of Plasmapheresis in Hemolysis, Elevated Liver Enzymes and Low Platelets (HELLP) Syndrome

**DOI:** 10.7759/cureus.35520

**Published:** 2023-02-27

**Authors:** Sobaan Taj, Mohamed Mujtaba, Brett Miller, Sowmaya Dandu, Christopher P Austin, Usman Ali Akbar, Harshavardhan Sanekommu, Mohammad A Hossain

**Affiliations:** 1 Internal Medicine, Jersey Shore University Medical Center, Neptune Township, USA; 2 Gastroenterology, Marshall University Joan C. Edwards School of Medicine, Huntington, USA; 3 Internal Medicine, St. George's University Medical School, True Blue, GRD; 4 Gastroenterology, North Shore University Hospital, New York City, USA; 5 Medicine, Hackensack Meridian School of Medicine, Nutley, USA

**Keywords:** liver transplantation in hellp, therapeutic plasmapheresis, liver disease of pregnancy, acute fatty liver of pregnancy, hellp syndrome

## Abstract

Hemolysis, elevated liver enzymes, and low platelet count (HELLP) syndrome is a rare abnormality comprising a series of symptoms that make up a syndrome. It usually happens during pregnancy or right after delivery. We describe a case of a 31-year-old female G4P2A2 (Gravida 4 Para 2 Abortions 2) who presented to the hospital for normal vaginal delivery but immediately postpartum developed HELLP syndrome. Acute fatty liver of pregnancy was a differential that the patient also met the criteria for. Her condition improved after starting her on plasmapheresis without considering hepatic transplantation. We emphasize distinguishing the overlap of symptoms between HELLP syndrome vs. acute fatty liver of pregnancy and the outcomes of plasmapheresis in managing HELLP syndrome without needing hepatic transplantation.

## Introduction

Hemolysis, elevated liver enzymes, and low platelet count (HELLP) syndrome is a rare but life-threatening condition that can happen during pregnancy or after delivery. It was first described by Weinstein in 1982 [1]. It is an acronym that represents a combination of clinical manifestations. This includes hemolysis, elevated liver enzymes, and low platelets [2]. HELLP syndrome is a significant cause of maternal and perinatal morbidity and mortality worldwide [3]. Incidence is about 0.5% to 0.9% and usually occurs between 28 and 37 weeks of gestation [4]. Almost 30% occur right after delivery. The nonspecific symptoms of this pathology make diagnosis difficult for practitioners. However, the diagnosis may occur when the usual clinical findings of severe preeclampsia are absent [2]. In order to make a differential diagnosis, such as acute fatty liver during pregnancy, imaging and laboratory tests are incredibly important [4]. We present a case of a 31-year-old female G4P2A2 (Gravida 4 Para 2 Abortions 2) who developed HELLP syndrome immediately postpartum and was managed with plasmapheresis with significant improvement. This case was previously presented as an abstract at the American College of Gastroenterology Annual Meeting of 2021.

## Case presentation

A 31-year-old Caucasian female G4P2A2 (Gravida 4 Para 2 Abortions 2), with gestational diabetes mellitus (DM) during her current pregnancy, presented to the hospital in active labor. She had no history of hypertension or preeclampsia. Blood pressure readings prior to presentation were within normal ranges. Her previous pregnancies were unremarkable, with no known complications. She had an uncomplicated vaginal delivery on the day of arrival at our hospital. Laboratory results during her hospital course are shown in Table 1.

**Table 1:** Labs and reference values throughout the patient's hospital course. BUN: Blood urea nitrogen; INR: International normalized ratio; LDH: Lactate dehydrogenase.

Labs and reference values	On Admission	24 hours postpartum	Labs 48 hours postpartum	Labs 96 hours postpartum
Hemoglobin (12-17.5g/dl)	14.1 g	7.2	8.4	8.2
White count (4.5-11 k/ul)	16.6	18	22	17.5
Platelets (140-450k/ul)	128	48	46	5l
Potassium (3.5-5.2mmol/dl)	4.5	4.5	3.9	3.4
Sodium (136-145mmol/dl)	134	130	137	138
Creatinine (0.44-1.0mg/dl)	0.78	0.77	1.5	1.4
BUN (5-25 mg/dl)	20	23	50	36
Alanine Aminotransferase (10-60IU/L)	186	3140	1345	162
Aspartate Aminotransferase (10-42 IU/L)	209	4770	2056	104
Total bilirubin (0.2-1.3 mg/dl)	0.3	10.3	15.6	8.1
INR (2-3 conventional anticoagulation).	1.04	1.43	1.6	1.0
Alkaline Phosphatase (38-126 IU/L)	175	164	101	74
Ammonia (11-55 umol/L)	N/A	144	74	33
LDH (120-250 IU/L)	N/A	6910	2500	546

**Figure 1:**
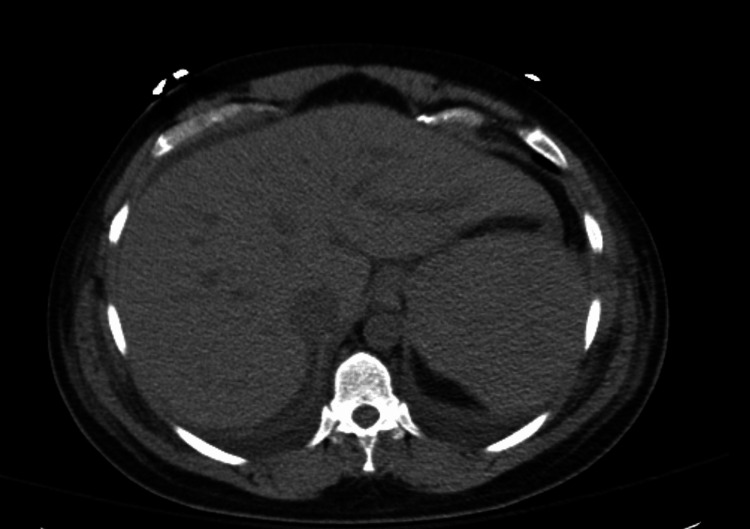
CT scan showing hepatomegaly with mild ascites.

Immediately postpartum, the patient had an elevation of blood pressure to 190/110 mmHg with right upper quadrant (RUQ) pain without a seizure, asterixis, confusion, or blurry vision. The patient was treated with IV magnesium for seizure prevention. Her blood pressure was managed with nifedipine for quicker relief and labetalol. The patient subsequently began to bleed from her IV lines. At this time, her liver enzymes had increased significantly. Further investigation revealed myoglobinuria, low haptoglobin, few schistocytes on a peripheral blood smear, acute kidney injury (AKI), elevated lipase, mildly elevated ammonia, elevated uric acid, and a disintegrin and metalloproteinase with a thrombospondin type 1 motif, and member 13 (ADAMTS13) (also known as von Willebrand factor cleaving protease) was 64.5% (normal range 60-130%). An abdominal CT scan revealed hepatomegaly with mild ascites, no subcapsular hematoma, and patent hepatic vasculature (Figure 1). At this time, she was diagnosed with HELLP syndrome Class 1 as her platelet count was below 50,000. The patient did, however, meet Swansea criteria for acute fatty liver of pregnancy. Her liver enzymes continued to rise, and she was actively coagulating. The patient was put on an emergent liver transplantation list for fears of worsening clinical status. However, she was started on plasmapheresis on postpartum day three for six sessions, where she showed significant clinical improvement. Her blood pressure normalized, abdominal pain resolved, and her liver enzymes progressively normalized without the requirement for liver transplantation.

## Discussion

With an incidence rate of about 0.5%-0.9%, HELLP syndrome is a rare yet life-threatening condition [4]. Due to variability in the literature, the diagnostic criteria of HELLP syndrome have been inconsistent [5]. However, the American College of Obstetricians and Gynecologists Task Force has now standardized the diagnostic criteria for hypertension in pregnancy [5]. The general criteria are as follows: at least two signs of hemolysis, elevated serum bilirubin, low serum haptoglobin, severe anemia unrelated to blood loss, elevated liver enzymes, and low platelets [5]. Other clinical signs include nausea, vomiting, a history of malaise, RUQ or epigastric pain, bleeding, etc. [5].

As a clinician, it can be challenging to differentiate HELLP syndrome from various other pathologies. These pathologies include but are not limited to viral hepatitis, Budd-Chiari syndrome, pyelonephritis, thrombocytopenic purpura/hemolytic uremic syndrome, and acute fatty liver of pregnancy (AFLP) [6]. As was the case in our patient, in addition to meeting HELLP syndrome criteria, she also met the diagnosing criteria for AFLP. The diagnosing criteria commonly used by clinicians are the Swansea criteria [7]. The diagnosis requires six or more of the following criteria: vomiting, abdominal pain, polydipsia/polyuria, encephalopathy, elevated bilirubin (>14 kmol/L), hypoglycemia (<72 mg/dL), elevated urate (>340 kmol/L), leukocytosis (11x10^6/L), ascites or bright liver on the sonogram, aminotransferase, alanine aminotransferase (>42 IU/L), ammonia (>27.5 mg/dl), creatinine >1.7 mg/dl, coagulopathy (prothrombin time >14 s, or activated partial thromboplastin time >34 s), and microvesicular steatosis on liver biopsy [7]. AFLP is a clinical diagnosis [8]. Although a liver biopsy is a gold standard in confirming an AFLP diagnosis, it is not commonly done as there is a need for urgent delivery and an ongoing risk of coagulopathy [8]. Following the diagnosis of HELLP syndrome, patients require prompt initiation of treatment. Treatments include corticosteroids to help improve laboratory abnormalities, magnesium sulfate for seizure prophylaxis, and antihypertensives if blood pressure is consistently more than 160/110 mmHg [9]. Often delivery itself can correct underlying abnormalities [9]. Due to blood abnormalities, 38-93% of patients with HELLP syndrome receive some blood product [9]. Plasma exchange therapy is an incredibly effective treatment for patients who were unresponsive to these initial interventions, as shown in limited reports and studies, to significantly decrease the maternal mortality rate [10]. Plasmapheresis has even been shown to be effective in patients who have organ failure [11]. Indications for liver transplantation are irreversible liver damage, liver necrosis, and uncontrollable bleeding [12]. The efficacy of plasmapheresis cannot be understated. One study found that overall survival in patients undergoing plasmapheresis therapy was similar between HELLP patients who received and did not receive a liver transplant [13]. Unnecessary liver transplantations could be avoided by treating the patient in a stepwise matter by treating with plasmapheresis before considering a transplant.

## Conclusions

HELLP syndrome is a rare, life-threatening condition. In this article, we emphasized the presentation and overlapping features of HELLP syndrome with acute fatty liver of pregnancy and the role of plasmapheresis in managing HELLP syndrome. We also found evidence from recent data that plasmapheresis decreases maternal mortality from 23.1% to 0%, further strengthening the role of plasmapheresis. We believe timely use of plasmapheresis in patients with HELLP syndrome will prevent unnecessary liver transplantation. We encourage more cases like this to be reported to develop better diagnostic criteria and therapeutic interventions to manage HELLP syndrome.
